# Bis[4-(dimethyl­amino)pyridinium] tribromidochloridodimethyl­stannate(IV)

**DOI:** 10.1107/S1600536808014669

**Published:** 2008-05-21

**Authors:** Kong Mun Lo, Seik Weng Ng

**Affiliations:** a Department of Chemistry, University of Malaya, 50603 Kuala Lumpur, Malaysia

## Abstract

The Sn^IV^ atom in the title salt, (C_7_H_11_N_2_)_2_[SnBr_3_(CH_3_)_2_Cl], lies on a center of inversion in a tetra­gonally compressed octa­hedron; two independent Br atoms share the same site as two independent chlorine atoms so that the anion effectively has one Cl and three Br atoms. The occupancies of the Br atoms are 0.721 (1) and 0.779 (1), and those of the Cl atoms are 0.279 (1) and 0.221 (1). The crystal structure involves N—H⋯halogen hydrogen bonds.

## Related literature

For the isostructural bis­(4-dimethyl­amino­pyridinium) dibromido­dichloro­dimethyl­stannate(IV), see: Lo & Ng (2008 ▶).
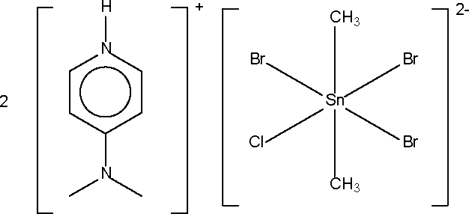


## Experimental

### 

#### Crystal data

(C_7_H_11_N_2_)_2_[SnBr_3_(CH_3_)_2_Cl]
*M* *_r_* = 670.29
Triclinic, 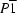

*a* = 7.3692 (2) Å
*b* = 8.6303 (1) Å
*c* = 9.5686 (2) Å
α = 96.902 (1)°
β = 106.546 (1)°
γ = 91.628 (1)°
*V* = 577.87 (2) Å^3^
*Z* = 1
Mo *K*α radiation
μ = 6.42 mm^−1^
*T* = 100 (2) K
0.35 × 0.15 × 0.10 mm

#### Data collection

Bruker SMART APEX diffractometer
Absorption correction: multi-scan (*SADABS*; Sheldrick, 1996 ▶) *T*_min_ = 0.212, *T*_max_ = 0.566 (expected range = 0.197–0.527)
7041 measured reflections
2623 independent reflections
2344 reflections with *I* > 2σ(*I*)
*R*_int_ = 0.022

#### Refinement

*R*[*F* ^2^ > 2σ(*F* ^2^)] = 0.021
*wR*(*F* ^2^) = 0.061
*S* = 1.07
2623 reflections
122 parameters
4 restraints
H-atom parameters constrained
Δρ_max_ = 0.89 e Å^−3^
Δρ_min_ = −0.63 e Å^−3^

### 

Data collection: *APEX2* (Bruker, 2007 ▶); cell refinement: *SAINT* (Bruker, 2007 ▶); data reduction: *SAINT*; program(s) used to solve structure: *SHELXS97* (Sheldrick, 2008 ▶); program(s) used to refine structure: *SHELXL97* (Sheldrick, 2008 ▶); molecular graphics: *X-SEED* (Barbour, 2001 ▶); software used to prepare material for publication: *publCIF* (Westrip, 2008 ▶).

## Supplementary Material

Crystal structure: contains datablocks global, I. DOI: 10.1107/S1600536808014669/sj2501sup1.cif

Structure factors: contains datablocks I. DOI: 10.1107/S1600536808014669/sj2501Isup2.hkl

Additional supplementary materials:  crystallographic information; 3D view; checkCIF report

## Figures and Tables

**Table:** 

Sn1—C1	2.131 (3)
Sn1—Br1	2.7240 (3)
Sn1—Br2	2.7234 (3)

**Table:** 

C1—Sn1—Br1	89.74 (7)
C1—Sn1—Br1^i^	90.26 (7)
Br1—Sn1—Br2	88.54 (1)
Br1—Sn1—Br2^i^	91.47 (1)

Symmetry code: (i) 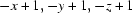.

**Table 2:** Hydrogen-bond geometry (Å, °)

*D*—H⋯*A*	*D*—H	H⋯*A*	*D*⋯*A*	*D*—H⋯*A*
N1—H1⋯X1	0.88	2.61	3.325 (2)	139
N1—H1⋯X2	0.88	2.83	3.475 (2)	132

Symmetry codes: .

**Fig. 1.**
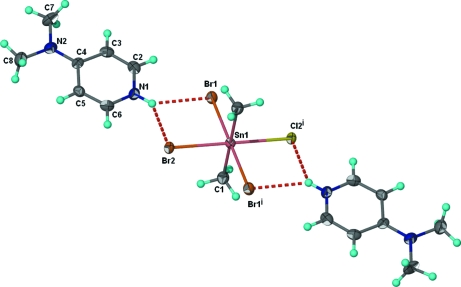
Thermal ellipsoid plot (Barbour, 2001) plot of [C7H11N2]2 [SnBr3Cl2(CH3)2] at the 70% probability level. Hydrogen atoms are drawn as spheres of arbitrary radius. The Sn atom lies on a center-of-inversion such that the two independent Br atoms are disordered with respect to the two independent Cl atoms. Symmetry code: i = 1 – x, 1 – y, 1 – z. Dashed lines denote hydrogen bonds.

## supplementary crystallographic information

### Comment

We have been investigating the reaction of organotin compounds with 4-dimethylpyridinium hydrobromide perbromide. In the previous study, this compound was reacted with dimethyltin dichloride to afford bis(4-dimethylpyridinium) dibromidodichloridodimethylstannate (Lo & Ng, 2008), whose. The halogens are in the expected 2:2 molar ratio. The bromine atoms are disordered with respect to the chlorine atoms. In the present study, the organotin reactant, chlorodimethyltin dimethyldithiocarbamate contains only one chlorine atom. The resulting stannate (Scheme I, Fig. 1) is the expected tribromidochloridodimethylstannate; the two salts are isostructural. N1—H1···X hydrogen bonds (X is a disordered mixture of Cl and Br; symmetry code: x, y, z) link the anions and cations, Fig 1, Table 2.

### Experimental

Chlorodimethyltin dimethyldithiocarbamate (1.54 g, 0.005 mol) and 4-dimethylpyridinium hydrobromide perbromide (1.81 g, 0.005 mol) were dissolved in a mixture of ethanol and chloroform (1:1) and the resulting mixture was refluxed for 15 minutes. Colorless crystals separated from the cool solution after several days.

### Refinement

Carbon-bound H-atoms were placed in calculated positions (C—H 0.95 to 0.98 Å) and were included in the refinement in the riding model approximation, with *U*(H) set to 1.2 to 1.5*U*_eq_(C). The ammonium H atom was similarly treated (N–H 0.88 Å).

The two indepedent chlorine atoms are disordered with respect to the bromine atoms, so that each halogen site is occupied by both a chlorine and a bromine. Restraints were applied so that at each site; the atoms were restrained to have the same anisotropic temperature factors. Without occupancy restraints, occupancies of the chlorine atoms refined to nearly 0.5 and the total number of bromine atoms to approximately 1.5. The sum of the occupancies were then restrained to these values and, in the final refinement, occupancies refined to Br1 0.721 (1), Br2 0.779 (1), Cl1 0.279 (1) and Cl2 0.221 (1). The final difference Fourier map was featureless.

### Crystal data

**Table:**

(C_7_H_11_N_2_)_2_[SnBr_3_(CH_3_)_2_Cl]	*Z* = 1
*M**_r_* = 670.29	*F*_000_ = 324
Triclinic, *P*1	*D*_x_ = 1.926 Mg m^−^^3^
Hall symbol: -P 1	Mo *K*α radiation λ = 0.71073 Å
*a* = 7.3692 (2) Å	Cell parameters from 3862 reflections
*b* = 8.6303 (1) Å	θ = 2.4–28.3º
*c* = 9.5686 (2) Å	µ = 6.42 mm^−^^1^
α = 96.902 (1)º	*T* = 100 (2) K
β = 106.546 (1)º	Prism, colorless
γ = 91.628 (1)º	0.35 × 0.15 × 0.10 mm
*V* = 577.87 (2) Å^3^	

### Data collection

**Table:**

Bruker SMART APEX diffractometer	2623 independent reflections
Radiation source: fine-focus sealed tube	2344 reflections with *I* > 2σ(*I*)
Monochromator: graphite	*R*_int_ = 0.022
*T* = 100(2) K	θ_max_ = 27.5º
ω scans	θ_min_ = 2.2º
Absorption correction: Multi-scan(SADABS; Sheldrick, 1996)	*h* = −9→8
*T*_min_ = 0.212, *T*_max_ = 0.566	*k* = −11→11
7041 measured reflections	*l* = −12→12

### Refinement

**Table:**

Refinement on *F*^2^	Secondary atom site location: difference Fourier map
Least-squares matrix: full	Hydrogen site location: inferred from neighbouring sites
*R*[*F*^2^ > 2σ(*F*^2^)] = 0.021	H-atom parameters constrained
*wR*(*F*^2^) = 0.061	*w* = 1/[σ^2^(*F*_o_^2^) + (0.0363*P*)^2^] where *P* = (*F*_o_^2^ + 2*F*_c_^2^)/3
*S* = 1.07	(Δ/σ)_max_ = 0.001
2623 reflections	Δρ_max_ = 0.89 e Å^−^^3^
122 parameters	Δρ_min_ = −0.63 e Å^−^^3^
4 restraints	Extinction correction: none
Primary atom site location: structure-invariant direct methods	

### Fractional atomic coordinates and isotropic or equivalent isotropic displacement parameters (Å^2^)

**Table:**

	*x*	*y*	*z*	*U*_iso_*/*U*_eq_	Occ. (<1)
Sn1	0.5000	0.5000	0.5000	0.01608 (8)	
Br1	0.50013 (5)	0.49555 (4)	0.78420 (3)	0.02259 (11)	0.7207 (11)
Br2	0.64681 (5)	0.21397 (3)	0.50475 (3)	0.02106 (10)	0.7793 (11)
Cl1	0.50013 (5)	0.49555 (4)	0.78420 (3)	0.02259 (11)	0.2793 (11)
Cl2	0.64681 (5)	0.21397 (3)	0.50475 (3)	0.02106 (10)	0.2207 (11)
N1	0.6543 (3)	0.1474 (3)	0.8573 (2)	0.0201 (5)	
H1	0.6158	0.2122	0.7921	0.024*	
N2	0.8485 (3)	−0.1517 (3)	1.1630 (2)	0.0221 (5)	
C1	0.2173 (4)	0.3966 (3)	0.4268 (3)	0.0218 (6)	
H1A	0.1467	0.4384	0.4941	0.033*	
H1B	0.2201	0.2829	0.4250	0.033*	
H1C	0.1550	0.4209	0.3276	0.033*	
C2	0.6702 (4)	0.1943 (3)	1.0009 (3)	0.0228 (6)	
H2	0.6368	0.2962	1.0297	0.027*	
C3	0.7329 (4)	0.0981 (3)	1.1038 (3)	0.0207 (5)	
H3	0.7430	0.1331	1.2039	0.025*	
C4	0.7837 (4)	−0.0549 (3)	1.0634 (3)	0.0169 (5)	
C5	0.7598 (4)	−0.0989 (3)	0.9112 (3)	0.0191 (5)	
H5	0.7883	−0.2008	0.8774	0.023*	
C6	0.6970 (4)	0.0029 (3)	0.8136 (3)	0.0215 (6)	
H6	0.6828	−0.0286	0.7123	0.026*	
C7	0.8693 (5)	−0.1024 (4)	1.3185 (3)	0.0307 (7)	
H7A	0.9483	−0.0041	1.3512	0.046*	
H7B	0.9297	−0.1832	1.3764	0.046*	
H7C	0.7440	−0.0869	1.3323	0.046*	
C8	0.9004 (4)	−0.3085 (3)	1.1218 (3)	0.0282 (6)	
H8A	0.9901	−0.3028	1.0639	0.042*	
H8B	0.7864	−0.3730	1.0631	0.042*	
H8C	0.9597	−0.3554	1.2108	0.042*	

### Atomic displacement parameters (Å^2^)

**Table:**

	*U*^11^	*U*^22^	*U*^33^	*U*^12^	*U*^13^	*U*^23^
Sn1	0.01588 (14)	0.01489 (13)	0.01660 (13)	0.00076 (9)	0.00352 (10)	0.00175 (9)
Br1	0.0347 (2)	0.01817 (17)	0.01694 (16)	0.00364 (13)	0.01007 (14)	0.00342 (12)
Br2	0.02735 (19)	0.01599 (15)	0.01872 (16)	0.00594 (12)	0.00403 (13)	0.00333 (11)
Cl1	0.0347 (2)	0.01817 (17)	0.01694 (16)	0.00364 (13)	0.01007 (14)	0.00342 (12)
Cl2	0.02735 (19)	0.01599 (15)	0.01872 (16)	0.00594 (12)	0.00403 (13)	0.00333 (11)
N1	0.0196 (12)	0.0193 (11)	0.0212 (11)	0.0008 (9)	0.0039 (9)	0.0067 (9)
N2	0.0207 (13)	0.0257 (12)	0.0189 (11)	0.0001 (10)	0.0034 (9)	0.0049 (9)
C1	0.0208 (14)	0.0214 (13)	0.0237 (13)	0.0021 (11)	0.0068 (11)	0.0044 (11)
C2	0.0200 (14)	0.0206 (13)	0.0273 (14)	0.0007 (11)	0.0074 (12)	−0.0001 (11)
C3	0.0172 (14)	0.0259 (14)	0.0184 (12)	−0.0033 (11)	0.0060 (11)	−0.0008 (10)
C4	0.0112 (12)	0.0215 (13)	0.0183 (12)	−0.0013 (10)	0.0036 (10)	0.0055 (10)
C5	0.0176 (14)	0.0169 (12)	0.0214 (13)	−0.0027 (10)	0.0044 (11)	0.0010 (10)
C6	0.0204 (15)	0.0257 (14)	0.0182 (12)	−0.0034 (11)	0.0063 (11)	0.0010 (10)
C7	0.0287 (17)	0.0428 (18)	0.0203 (13)	0.0058 (14)	0.0037 (12)	0.0110 (13)
C8	0.0267 (16)	0.0253 (15)	0.0320 (15)	0.0027 (12)	0.0053 (13)	0.0095 (12)

### Geometric parameters (Å, °)

**Table:**

Sn1—C1	2.131 (3)	C1—H1A	0.9800
Sn1—Br1	2.7240 (3)	C1—H1B	0.9800
Sn1—Br2	2.7234 (3)	C1—H1C	0.9800
N1—C6	1.342 (3)	C2—H2	0.9500
N1—C2	1.356 (3)	C3—H3	0.9500
N2—C4	1.338 (3)	C5—H5	0.9500
N2—C8	1.456 (4)	C6—H6	0.9500
N2—C7	1.460 (3)	C7—H7A	0.9800
C2—C3	1.354 (4)	C7—H7B	0.9800
C3—C4	1.421 (4)	C7—H7C	0.9800
C4—C5	1.419 (3)	C8—H8A	0.9800
C5—C6	1.353 (4)	C8—H8B	0.9800
N1—H1	0.8800	C8—H8C	0.9800
			
C1—Sn1—C1^i^	180.0	Sn1—C1—H1C	109.5
C1—Sn1—Br1	89.74 (7)	H1A—C1—H1C	109.5
C1—Sn1—Br1^i^	90.26 (7)	H1B—C1—H1C	109.5
Br1—Sn1—Br1^i^	180.0	C3—C2—H2	119.5
Br1—Sn1—Br2	88.54 (1)	N1—C2—H2	119.5
Br1—Sn1—Br2^i^	91.47 (1)	C2—C3—H3	119.7
Br2—Sn1—Br2^i^	180.0	C4—C3—H3	119.7
C6—N1—C2	120.5 (2)	C6—C5—H5	119.7
C4—N2—C8	121.9 (2)	C4—C5—H5	119.7
C4—N2—C7	120.5 (2)	N1—C6—H6	119.4
C8—N2—C7	117.6 (2)	C5—C6—H6	119.4
C3—C2—N1	120.9 (2)	N2—C7—H7A	109.5
C2—C3—C4	120.6 (2)	N2—C7—H7B	109.5
N2—C4—C5	122.2 (2)	H7A—C7—H7B	109.5
N2—C4—C3	121.8 (2)	N2—C7—H7C	109.5
C5—C4—C3	116.0 (2)	H7A—C7—H7C	109.5
C6—C5—C4	120.6 (2)	H7B—C7—H7C	109.5
N1—C6—C5	121.3 (2)	N2—C8—H8A	109.5
C6—N1—H1	119.7	N2—C8—H8B	109.5
C2—N1—H1	119.7	H8A—C8—H8B	109.5
Sn1—C1—H1A	109.5	N2—C8—H8C	109.5
Sn1—C1—H1B	109.5	H8A—C8—H8C	109.5
H1A—C1—H1B	109.5	H8B—C8—H8C	109.5
			
C6—N1—C2—C3	1.4 (4)	C2—C3—C4—N2	179.1 (3)
N1—C2—C3—C4	0.0 (4)	C2—C3—C4—C5	−1.5 (4)
C8—N2—C4—C5	0.5 (4)	N2—C4—C5—C6	−178.8 (3)
C7—N2—C4—C5	−179.4 (3)	C3—C4—C5—C6	1.7 (4)
C8—N2—C4—C3	179.9 (2)	C2—N1—C6—C5	−1.2 (4)
C7—N2—C4—C3	0.0 (4)	C4—C5—C6—N1	−0.4 (4)

Symmetry codes: (i) −*x*+1, −*y*+1, −*z*+1.

### Hydrogen-bond geometry (Å, °)

**Table:**

*D*—H···*A*	*D*—H	H···*A*	*D*···*A*	*D*—H···*A*
N1—H1···X1	0.88	2.61	3.325 (2)	139
N1—H1···X2	0.88	2.83	3.475 (2)	132
